# A Comparative Study on Novel-Assisted Extraction Techniques for Retrieving Protein from *Moringa oleifera* Seeds

**DOI:** 10.3390/foods14173046

**Published:** 2025-08-29

**Authors:** Paul Ndubuisi Anyiam, Pipat Tangjaidee, Wanli Zhang, Saroat Rawdkuen

**Affiliations:** 1 Innovative Food Science and Technology Program, School of Agro-Industry, Mae Fah Luang University, Chiang Rai 57100, Thailand; 6671401002@lamduan.mfu.ac.th; 2 Department of Biochemistry, College of Natural Science, Michael Okpara University of Agriculture, Umudike P.M.B 7262, Abia State, Nigeria; 3 Division of Food Science and Technology, Faculty of Agro-Industry, Chiang Mai University, Chiang Mai 50100, Thailand; pipat.t@cmu.ac.th; 4 College of Food Science and Technology, Hainan University, Haikou 570228, China; zwl@hainanu.edu.cn; 5 Unit of Innovative Food Packaging and Biomaterials, School of Agro-Industry, Mae Fah Luang University, Chiang Rai 57100, Thailand

**Keywords:** emerging extraction technologies, plant-based, moringa seed protein, in vitro digestibility

## Abstract

*Moringa oleifera* seeds are rich in protein, yet their potential as plant-based protein in food remains underutilized. This study evaluated the extraction efficiency, composition, and techno-functional properties of moringa seed protein isolate (MSPI) using enzyme-assisted (EAE), ultrasonic-assisted (UAE), and microwave-assisted extraction (MAE) methods, compared to conventional alkaline extraction (CE). EAE was performed with viscozyme (2%, pH 8, 50 °C, 2 h) and papain (1%, pH 7, 50 °C, 1 h), UAE at 40% amplitude (20 kHz, 20 min), and MAE at 800 W (50 °C, 90 s). All methods significantly improved extraction yield (14.60–30.08%), protein content (80.47–86.61%), solubility (40.78–60.09% at pH 10), and techno-functional properties over CE. However, MAE slightly reduced solubility. Phytates (0.83–0.49 g/100 g) and trypsin inhibitor activity significantly decreased (4.48–1.92 U/mg). In vitro protein digestibility improved (*p* < 0.05) across all samples (88.11–93.81%), with hydrolysis patterns supporting the enhanced digestibility. Structural modifications were indicated by altered surface hydrophobicity and thermal properties. SDS-PAGE showed consistent major protein bands at 17, 25, and 48–63 kDa, with EAE showing reduced intensity at ~63 kDa. While UAE and MAE achieved high protein yield and purity, EAE offered the best balance of functionality and digestibility, making it the most promising method for producing high-quality MSPI. These findings are relevant for guiding the selection of extraction methods for MSPI recovery for food applications.

## 1. Introduction

The growing demand for sustainable, functional, and nutritionally rich protein sources has driven significant interest in plant-based proteins as viable alternatives to animal-derived proteins [1]. Among the many plant-based protein sources, *Moringa oleifera* seeds are gaining growing interest due to their exceptional nutritional profile, including a high protein content (28–51%), a complete range of essential amino acids, and a variety of health-promoting properties such as antioxidant, antimicrobial, and anti-cancer effects [2,3]. The essential-to-total amino acid ratio (31.17%) recently reported for moringa seed, fulfills the minimum requirements for children (26.0%) and adults (15.0%) and its total aromatic amino acids are closely similar to that of beef and egg protein [4]. Although moringa seeds are rich in protein, their use in modern food processing is limited. Traditionally, various parts of the plant (leaves, stem, roots, and seeds) have been used in folk medicine in African and Asian communities for the treatment of various diseases [5]. Due to their balanced amino acid profile, moringa seed proteins hold promise as functional ingredients and potential alternatives to conventional proteins in human diets. However, their utilization in food production is still very low and currently only a few value-added products or ingredients derived from this source are currently available in the commercial market. This is likely due to their poor sensory attributes and the presence of anti-nutritional factors (ANFs) [4,6]. Therefore, understanding how different extraction methods influence the functional properties and the reduction in ANFs of moringa seed proteins is crucial for unlocking its full potential as a high-value food ingredient.

The main challenge in extracting plant proteins is to choose the appropriate extraction technique, due to the intricate structure of the plant cell wall [7]. Traditional extraction methods, such as alkaline solubilization, are widely used to isolate plant proteins in many studies. Although effective in extracting protein, this method often leads to loss of functional properties and insufficient removal of ANFs, such as phytates, tannins, and trypsin inhibitors [7,8]. These ANFs can negatively impact protein quality by reducing digestibility, stability, and the application in food formulations. To overcome these limitations, novel-assisted extraction techniques, such as enzyme-assisted extraction (EAE), ultrasound-assisted extraction (UAE), and microwave-assisted extraction (MAE), have gained attention in recent years [7,8,9]. These techniques are categorized as novel due to their relatively recent adoption in food processing, as well as their potential to enhance extraction efficiency, improve the quality of the extracted proteins, and minimize degradation caused by harsh processing conditions.

The efficiency and suitability of these novel extraction technologies vary depending on the plant matrix and the nature of the target protein [8]. Recent studies have explored the application of different novel extraction methods in various plant proteins, such as soy [10], pea protein [11], and common bean [12]. However, limited data exist on their impact on moringa seed protein recovery. A study carried out by Fatima et al. [6] investigated UAE for enhancing protein yield and functional properties from *Moringa* seeds. While this study highlights the potential of UAE in protein recovery from moringa seeds, comprehensive comparative studies evaluating the efficiency of other novel techniques are lacking. Moreover, limited research has examined the impact of novel extraction methods on ANFs in plant protein isolates, as most existing studies have largely overlooked this aspect. Therefore, this study aimed to investigate and compare the effects of EAE, UAE, and MAE methods with conventional alkaline extraction (CE) on protein recovery from moringa seeds. Specifically, it evaluates protein yield, in vitro protein digestibility, residual ANFs, thermal stability using differential scanning calorimetry, and techno-functional properties of extracted protein. The findings of this study are expected to contribute to the development of efficient extraction protocols that enhance the quality and applicability of moringa seed protein isolate (MSPI) in food systems.

## 2. Materials and Methods

### 2.1. Materials

Fresh and mature moringa seeds were obtained from a local farm in Mueang district, Chiang Rai province, Thailand. All chemicals and reagents used in the study, including, Tris(hydroxymethyl)aminomethane (THAM), methyl red, sodium dodecyl sulfate (SDS), glacial acetic acid, methanol, and Coomassie blue R-250, were purchased from RCI LabScan Ltd., Bangkok, Thailand. The enzymes used in the experiment, pancreatin (EC-232-468-9, CAS: 8049-47-6), pepsin (EC 3.4.23.1; CAS: 9001-75-6), trypsin (EC-232-650-8; CAS: 9002-07-7), viscozyme L (a multi-enzyme complex), papain (EC-232-627-2; CAS:9001-73-4), and bovine serum albumin (BSA), were purchased from Sigma-Aldrich Chemical Co., (St. Louis, MO, USA). The pre-stained protein marker was supplied by Bio-Helix Co., Ltd., Taipei City, Taiwan. All other chemicals and reagents were of analytical grade. Commercial soy protein isolate (C-SPI), used as a reference in certain analyses, was purchased from a local supermarket in the same location.

### 2.2. Sample Preparation

The obtained moringa seeds were manually removed from their pods and rinsed with tap water to eliminate surface dirt. The seeds were visually inspected, and any defective or immature seeds were discarded. Dehulling was performed by hand to remove the seed coats, after which the seed kernels were dried in a hot air oven (Memmert-A55/33, Schwabach, Germany) at 45 °C for 36 h. The dried seeds were then ground using an electric blender (RT-04A, Rong Tsong Tech. Co., Taichung City, Taiwan) and sieved through a 2 mm mesh to obtain a fine powder. The powder was defatted using n-hexane (1:3 *w*/*v*) by stirring for 3 h at 25 °C [13]. After decanting the hexane, the defatted flour was air-dried under a fume hood at ambient temperature (25 °C) with an approximate airflow rate of 0.5 m/s for 12 h to ensure complete solvent evaporation. The protein content was determined as outlined in Section 2.4. The defatted sample was packed in a resealable polyethylene zip-lock bag, and stored at 4 °C until use for protein extraction.

### 2.3. Protein Extraction Procedure

#### 2.3.1. Alkaline Extraction

Moringa seed protein isolate (MSPI) was prepared following the method of Illingworth et al. [13], with slight modifications. Briefly, the defatted moringa seed sample was mixed with deionized water at 1:10 (*w*/*v*) ratio. The pH was adjusted to 9.5 using 1.0 M NaOH, and the mixture was stirred with a magnetic stirrer (550 rpm) at 25 °C for 2 h. Subsequently, centrifugation was performed at 10,000× *g* for 10 min at 4 °C (Universal 320R, Hettich, Tuttlingen, Germany) and the supernatant was collected. The pH of the supernatant was adjusted to 4.5 using 1.0 M HCl, followed by continuous stirring for 1 h at 25 °C (room temperature) to precipitate the proteins. The protein precipitate was recovered by centrifugation (10,000× *g*, 10 min, 4 °C), resuspended in distilled water (1:5 *w*/*v*), and adjusted to pH 7. The resulting suspension was freeze-dried (Beta LSCbasic, Labconco, Kansas City, MO, USA). The final product was packed in a low-density polyethylene zip-lock bag, labeled as conventionally extracted protein (CE), and stored at −20 °C until further analysis.

#### 2.3.2. Enzyme-Assisted Extraction

The enzyme-assisted extraction was conducted with a sequential enzymatic pretreatment prior to the alkaline extraction of moringa seed proteins, following the method described by Yeasmin et al. [12] with slight modifications. The defatted sample was dispersed in deionized water (1:10 *w*/*v*), and the pH was adjusted to 5.5 (optimal for viscozyme L) using 1.0 M HCl. After stirring for 10 min at 25 °C, viscozyme L (2% *w*/*w*, enzyme/sample, activity: 100 FBG/ug) was added, and the mixture was incubated at 50 °C for 3 h with shaking (50 rpm). Thereafter, the pH was adjusted to 7.0 with 1.0 M NaOH, followed by the addition of papain (1% *w*/*w*, activity: 2.9 U/mg). Incubation continued at 50 °C for another 1 h, after which enzymes were inactivated at 90 °C for 10 min. Then subsequent protein extraction followed the alkaline procedure detailed in Section 2.3.1. The extracted protein was freeze-dried, packed in a low-density polyethylene zip-lock bag, labeled as enzyme-extracted protein (EAE), and stored at −20 °C until further analysis.

#### 2.3.3. Ultrasonic-Assisted Extraction

Ultrasonic-assisted extraction was carried out following the method described by Fatima et al. [6], with slight modifications. The defatted sample was dispersed in deionized water at a solid-to-liquid ratio of 1:10 (*w*/*v*). The pH of the dispersion was adjusted to 9.5 using 1 M NaOH to enhance protein solubility. The mixture was treated with a Vibra-Cell ultrasonic processor (VCX500, Sonics & Materials, Inc., Newtown, CT, USA) equipped with a 13 mm probe, operated at 40% amplitude and 20 kHz for 20 min at 25 °C, with a pulsed cycle of 5 s on and 5 s off. The ultrasound probe was submerged at 1/3 of the liquid volume. In order to avoid overheating, an ice bath was used to cool the sample during ultrasonication. After sonication, the protein extraction proceeded through the isoelectric precipitation and neutralization steps outlined in Section 2.3.1. Extractions were performed in triplicate and the samples were lyophilized. The final product was packed in a low-density polyethylene zip-lock bag, labeled as ultrasonic-extracted protein isolate (UAE), and stored at −20 °C until further analysis. The selected ultrasonic conditions were based on previously reported optimized conditions for efficient protein recovery [6,7].

#### 2.3.4. Microwave-Assisted Extraction

Microwave-assisted extraction was performed using a laboratory-grade microwave system (THOS X/UP system, Milestone Srl, Sorisole, Italy) following the method described by Ajayi et al. [14], with slight modifications. The defatted sample was mixed with deionized water at a ratio of 1:10 (*w*/*v*). The pH of the mixture was adjusted to 9.5 using 1 M NaOH. The sample was then stirred with a magnetic stirrer (MAG HS 7, IKA, Staufen, Germany) at 80 rpm for 30 min at 25 °C (room temperature), before being subjected to microwave extraction at 800 W for 90 s. Afterwards, the mixture was allowed to cool to room temperature (25 ± 2 °C) before the isoelectric precipitation and neutralization steps described in Section 2.3.1. The selected microwave conditions were based on optimized parameters reported in the literature [7]. All extractions were performed in triplicate. The obtained sample was freeze dried, labeled microwave-extracted moringa seed protein isolate (MAE), and stored in a resealable low-density polyethylene zip-lock bag at −20 °C until further assay.

#### 2.3.5. Determination of Protein Yield and Recovery

All analyses were compared against the conventionally extracted moringa seed protein (CE) and, where applicable, C-SPI. The protein extraction yield (%) and protein recovery rate (%) of each extraction method were calculated using Equations (1) and (2), respectively [14]. The protein content of each isolate was analyzed using the Kjeldahl method, as outlined in Section 2.4.1. Equations (1) and (2) are as follows:

(1) $$\mathrm{Protein}\;\mathrm{extraction}\;\mathrm{yield}\;(\%)=\frac{Weight\;of\;isolate\;(g)}{Initial\;weight\;of\;DMF\;(g)}\times 100$$

(2) $$\mathrm{Protein}\;\mathrm{recovery}\;\mathrm{rate}\;(\%)=\frac{WI\times PI}{WS\times PS}\times 100$$

where ‘WI’ is the weight (g) of isolated protein on a dry weight basis, ‘WS’ is the initial weight (g) of defatted moringa seed flour, ‘PI’ means protein content (%) of isolated protein, ‘PS’ means protein content (%) in seed flour, and DMF is the defatted moringa seed flour.

### 2.4. Chemical Analysis

#### 2.4.1. Proximate Composition

The proximate analyses of each protein isolate were conducted in triplicate following the official methods of the Association of Official Analytical Chemists (AOAC) [15]. Ash was measured after sample incineration at 550 °C using a gravimetric method (923.03), while the moisture content was determined by oven-dry method by heating the solid samples up to 105 °C until constant weight, and the dry weight was calculated gravimetrically (AOAC-950.46). Protein content was determined using the Kjeldahl method (AOAC 954.01) with a nitrogen-to-protein conversion factor of 6.25, while fat content was analyzed by the Soxhlet extraction method (AOAC 960.39) using petroleum ether. Results were reported as percentages based on the dry weight of the samples.

#### 2.4.2. Determination of Phytic Acid Content

The phytic acid content in each MSPI sample was determined using a method adapted from Reddy et al. [16]. For analysis, 2 g of the protein isolate were immersed in 100 mL of 2% hydrochloric acid and allowed to stand at 25 °C for 5 h. After extraction, the solution was filtered through Whatman No. 1 filter paper. A 25 mL portion of the clear filtrate was transferred into a conical flask, followed by the addition of 5 mL of a 0.3% potassium thiocyanate solution. This mixture was then titrated with a freshly prepared 1.05% (*w*/*v*) ferric chloride (FeCl_3_) solution. The endpoint of the titration was marked by the appearance of a stable brownish-yellow coloration that remained unchanged for a minimum of 5 min. The phytic acid concentration was calculated using Equation (3).

(3) $$\mathrm{Concentration}\;\mathrm{of}\;\mathrm{phytate}\;(g/100\;g)=\;\frac{Titre\;value\times 0.1081}{Sample\;weight\;(g)}$$

#### 2.4.3. Determination of Trypsin Inhibitor Activity

Trypsin inhibitor activity (TIA) was determined according to the method described by Wintersohle et al. [17]. Briefly, each protein isolate, weighing 1g, was extracted in 50 mL of 10 mM NaOH (80 rpm, 25 ± 2 °C, 3 h). The extract was diluted to achieve 30–70% trypsin inhibition. For the assay, 1 mL of extract was mixed with 2.5 mL substrate and 1 mL trypsin solution, incubated at 37 °C for 10 min, and stopped with 1 mL of 30% acetic acid. After centrifugation (10,000× *g*, 5 min), absorbance at 410 nm was measured (A410S). The reference absorbance (A410R) was measured by replacing the sample extract with water (absence of inhibitors). Reagent blanks for the sample (A410SB) and reference (A410RB) were prepared by adding acetic acid before the trypsin solution to inactivate the enzyme. One trypsin unit (TU) corresponds to an absorbance increase of 0.02 at 410 nm. TIA was expressed as TIU per mg protein (dw) and calculated using Equation (4).

(4) TIU/mgprotein = [(A410R−A410RB)−(A410S−A410SB)×100×mLdilutedproteinextract]mgsamplepermLdilutedproteinextract ×protein content

#### 2.4.4. Color Change Analysis

A Hunter-Lab instrument (Cox-2339, Reston, VA, USA) was used to measure the impact of extraction method on color change in MSPI. Firstly, colorimeter calibration was performed using white tiles. Then, the L* (lightness: 0–100), a* (redness or greenness), and b* (yellowness or blueness) values of each sample were measured in triplicate at three randomly chosen spots. The measured values were applied to compute the whiteness index (WI), yellowness index (YI), and color difference (ΔE) for each isolate using Equations (5), (6), and (7), respectively [7]. The changes in color were compared to the color parameters of commercial soy protein isolate (C-SPI).

(5) $$\mathrm{Whiteness}\;\mathrm{index}\;=\;100-\sqrt{(100-{L)}^{2}+{\left(a\right)}^{2}+{\left(b\right)}^{2}}$$

(6) $$\mathrm{Yellowish}\mathrm{index}\;=\frac{142.86b}{L}$$

(7) $$\Delta E^{*}=\sqrt{[(∆{L^{*})}^{2}+(∆{a^{*})}^{2}+(∆{b^{*})}^{2}}]$$

#### 2.4.5. SDS-PAGE

Each moringa seed protein isolate, weighing 2 g, was extracted with 18 mL of 5% SDS, homogenized at 10,000× *g* for 2 min (Ultra-Turrax T18, IKA, Wilmington, NC, USA), and heated at 85 °C for 1 h in a water bath to extract the proteins. After cooling to 25 °C (ambient temperature), the mixture was centrifuged at 10,000× *g* for 5 min, and protein content in the supernatant was measured using the Biuret method. SDS-PAGE was conducted by following the method described by Laemmli [18], using a 12% separating gel and 4% stacking gel. Protein samples (4 mg/mL) were mixed 1:1 (*v*/*v*) with loading buffer. For non-reducing conditions, the buffer contained 0.5 M Tris–HCl (pH 6.8), 0.5% bromophenol blue, 10% glycerol, and 2% SDS: β-mercaptoethanol was added for reducing conditions. Samples were heated at 95 °C for 5 min and then cooled to room temperature (25 ± 2 °C). A 4 μL aliquot (~15 μg protein) was loaded onto 4–12% gradient gels and electrophoresed at 15 mA per gel using a Mini-PROTEAN Tetra Cell (Bio-Rad Lab, Inc., Richmond, CA, USA). Gels were stained overnight with Coomassie Brilliant Blue R-250 under gentle shaking (50 rpm), followed by de-staining with methanol–acetic acid–water solutions I and II, and then dried and imaged under white light. A molecular weight marker ranging from 11 to 245 kDa was used to estimate protein sizes.

### 2.5. Techno-Functional Properties of Moringa Seed Protein Isolate

#### 2.5.1. Water and Oil Holding Capacity

Water holding capacity (WHC) was determined following the method of Illingworth et al. [13], with minor modifications. Each protein isolate sample weighing 1 g was mixed with 10 g of distilled water in a pre-weighed centrifuge tube. The mixture was vortexed for 2 min and allowed to stand at 25 °C for 1 h. It was then centrifuged at 5000× *g* for 10 min. The supernatant was carefully discarded without disturbing the residue, and the remaining residue in the tube was re-weighed to determine the amount of water retained. WHC was calculated using Equation (8). The same procedure was followed to determine the oil holding capacity (OHC), using 10 g of commercial sunflower oil instead of water. The retained oil was used to calculate OHC using Equation (9). Equations (8) and (9) are as follows:

(8) $$\mathrm{WHC}\;(g/g)=\frac{W2-W1}{Wo}\times 100$$

(9) $$\mathrm{OHC}\;(g/g)=\frac{W2-W1}{Wo}\times 100$$

where ‘W2’ is the weight of centrifuge tube, sample, and absorbed water/oil (g); W1 is the weight of centrifuge tube and sample (g); and ‘Wo’ is the weight of sample (g).

#### 2.5.2. Emulsification Properties

The emulsifying activity index (EAI) and emulsifying stability index (ESI) of each extracted MSPI were investigated to determine the emulsification properties using the method described by Constantino and Garcia-Rojas [19], with slight adjustments. To determine the EAI, 15 mL of a protein solution (prepared by dissolving 0.5 g of protein in 50 mL of distilled water at pH 7.0) was mixed with 5 mL of refined sunflower oil and homogenized using an IKA ULTRA-TURRAX T18 (IKA-Werke GmbH & Co. KG, Staufen, Germany) at 6500× *g* for 2 min. Next, 50 μL of the emulsion was combined with 5 mL of a sodium dodecyl sulfate (SDS) solution (0.1 g per 100 mL). The absorbance of the mixture was then measured immediately at 500 nm using a spectrophotometer (V-Visible-BIC99877, Biochrom/Libra, Cambridge, UK). To measure ESI, the same methodology was repeated but the absorbance was measured after 30 min of incubation at room temperature (25 °C). EAI and ESI were calculated using Equations (10) and (11), respectively:

(10) $$\mathrm{EAI}\;(m^{2}/g)=\frac{2\times 2.303\times DF\times A0}{C\times \theta \times \varphi \times 10,000}\times 100$$

(11) $$\mathrm{ESI}\;(\%)=\frac{Emulsifying\;capacity\;at\;30\;min}{Emulsifying\;capaity\;at\;0\;min}\times 100$$

where DF indicates the dilution factor for emulsion, which is 100; ‘Ao’ is the absorbance at zero min; C is the concentration of the initial sample, which is 0.1 g/mL; ‘θ’ is the length of the light path in the cuvette (0.01 m); and φ is the fraction of oil in the initial emulsion, which is 0.25.

#### 2.5.3. Foaming Properties Determination

Foam capacity (FC) and Foam stability (FS) of each MSPI were assessed using a method described by Illingworth et al. [13]. A 15 mL protein solution (0.50 g dissolved in 50 mL of distilled water, pH 7.0) was stirred for 45 min using a magnetic stirrer, then homogenized (IKA ULTRA-TURRAX T18, Staufen, Germany) at 6500× *g* for 2 min. The resulting mixture was immediately transferred into a 50 mL graduated cylinder with the help of a spatula. Foam volume was recorded immediately to assess FC and again after 30 min to evaluate FS. FC and FS were calculated using Equations (12) and (13), respectively.

(12) $$\mathrm{FC}\;(\%)=\frac{Volume\;of\;foam}{Volume\;of\;protein\;solution}\times 100$$

(13) $$\mathrm{FS}\;(\%)=\frac{\left(Volume\;of\;foam\;after\;30\;min\;holding\right)}{Initial\;volume\;of\;foam}\times 100$$

#### 2.5.4. Protein Solubility Assay

The solubility of each isolated moringa seed protein was evaluated across a pH range of 2.0 to 10.0 and compared with that of commercial soy protein isolate, following the method described in our previous study [4]. Bovine serum albumin was used to generate the standard curve (Y = 0.0537x; R^2^ = 0.9989). The percentage solubility was calculated by using Equation (14) and plotted against the corresponding pH values.

(14) $$\%\;\mathrm{Protein}\mathrm{solubility}=\frac{Soluble\;protein\;(g)}{Total\;protein\;in\;sample\;(g)}\times 100$$

#### 2.5.5. Surface Hydrophobicity Determination

The effect of the extraction method on the surface hydrophobicity (Ho) of MSPI was evaluated to assess the involvement of hydrophobic regions, following the procedure described by Hegde et al. [20] with slight modifications. A mixture of 200 μL of 1 mg/mL bromophenol blue (BPB) and 1 mL of protein solution (5 mg/mL) was stirred thoroughly for 10 min and then centrifuged at 5590× *g* for 15 min. The absorbance of the supernatant was measured at 595 nm using a UV–Vis spectrophotometer (UV-Visible-BIC99877, Biochrom/Libra, Cambridge, UK). Deionized water was used as a control in place of the protein solution. All analyses were performed in triplicate, and surface hydrophobicity was calculated according to Equation (15).

(15) $$\mathrm{Ho}\;(\mathrm{BPB}\mathrm{bound})\;=200\times \frac{Absorbance\;of\;control-Absorbance\;of\;sample}{Absorbance\;of\;control}\mu g$$

### 2.6. Determination of Thermal Properties

The thermal characteristics of MSPI were analyzed using a differential scanning calorimeter (DSC 3+, Mettler Toledo, Greifensee, Switzerland) following the sample preparation method described by Wintersohle et al. [17]. Protein isolates were dispersed in deionized water at 20% (*w*/*v*), stirred for 2 h, and allowed to hydrate overnight. The DSC was calibrated for temperature and heat capacity using aluminum standards. Samples of 10 mg protein suspension were sealed in standard aluminum pans, with an empty pan serving as the reference. The pans were heated from 20 to 115 °C at a consistent rate of 10 K/min during both heating and cooling. Transition enthalpy (ΔH) and denaturation temperature (Td) were determined from the thermograms using the instrument’s software.

### 2.7. In Vitro Protein Digestibility Assay

In vitro protein digestibility (IVPD) of each of the extracted MSPIs was evaluated in two stages using the sequential digestion procedure described in our previous report [4]. Sampling for pepsin digestion was conducted at 0, 30, 60, and 120 min of digestion, while for pancreatin digestion samples were collected at 150, 180, and 240 min. The percentage of IVPD was calculated using Equation (16) [21]. Polypeptide hydrolysis was assessed throughout the 240 min digestion period using SDS-PAGE, at each sampling interval.

(16) $$\mathrm{IVPD}\;(\%)\;=\frac{Initial\;Protein\;content-Protein\;content\;after\;digestion}{Initial\;protein\;content}\times 100$$

### 2.8. Surface Morphology Determination

The morphological characteristics of the MSPIs obtained from different extraction methods were examined using a scanning electron microscope (MIRA4; Tescan, Brno, Czech Republic), following the procedure described by Hegde et al. [20] with modifications. The freeze-dried samples were mounted on carbon-coated aluminum stubs using a metallic carbon adhesive, and subsequently sputter-coated with a 10 nm gold layer at a current of 10 mA for 45 s. The surface morphology of the samples was examined under accelerating voltage of 10 keV, a working distance of 10.52 mm, and a field of view of 372 µm. Micrographs were captured at 500× and 5000× magnifications at multiple randomly selected fields per sample, to cover the general structure.

### 2.9. Comparative Analysis

A comparative analysis was conducted using a radar plot to evaluate the efficiency of different extraction methods for protein recovery from moringa seed. All measured parameters were compared against commercial soybean protein isolate. For effective visualization each attribute was normalized, and the data for ANFs (phytate and TIA) were inverted and lower values were scored higher to reflect their desirable reduction. Each extraction method was scored from 1 (lowest) to 5 (highest) based on individual parameters, and the total scores were summed for ranking. Key criteria for scoring included protein extraction yield, protein content (i.e., % purity), in vitro digestibility, levels of ANFs, protein solubility, and techno-functional properties, which were summed together and ranked accordingly. Additional factors such as yellowness and thermal stability were also considered in the overall assessment.

### 2.10. Statistical Analysis

Protein extraction and all quantitative measurements were performed in triplicate to guarantee the reliability and accuracy of the findings. The data obtained were analyzed using analysis of variance (ANOVA) with SPSS software (version 20.0, SPSS Inc., Chicago, IL, USA). Results are expressed as mean ± standard deviation. Mean comparisons were conducted using the least significant difference (LSD) post hoc test, with significance determined at *p* < 0.05.

## 3. Results and Discussion

### 3.1. Effect of Extraction Methods on Protein Yield and Proximate Composition

Protein accessibility in plant cells is limited by the cell wall; thus, disruption of the plant cell wall is a crucial step to enhance extraction yield and efficiency. The extraction yield and proximate composition of the MSPIs extracted using different novel-assisted methods are presented in Table 1. All extraction techniques significantly (*p* < 0.05) improved both protein yield (14.60–30.08%) and recovery (37.47–78.43%) compared to the CE method. UAE recorded the highest improvement in yield (30.08%), while EAE had the lowest improvement (21.03%). This is attributed to the acoustic cavitation and shockwave effects of UAE that enhance cell wall disruption and mass transfer [7]. Fatima et al. [6] reported a yield increase from 15.65 to 39.12% for *Moringa oleifera* seeds using UAE, which aligns with our results. Similar yield enhancement was also recorded in ultrasonicated sesame seed protein compared to other novel extraction methods applied [22]. According to the authors, ultrasound treatment promotes bubble formation and the resulting cavitation and shear forces produced upon bubble collapse disrupt cell walls, increasing exposed surface area and the protein–solvent interface, thereby facilitating more protein release. Application of MAE also improved the protein yield of the MSPI compared to CE. This is attributed to the rapid heating effect and cell wall disruption by microwave energy, which promotes solvent permeability and facilitates the release of intracellular proteins. A similar result was reported by Coutinho et al. [11] for microwave-extracted pigeon pea protein, where a higher microwave power of 900 W for 120 s increased protein yield, whereas a lower power of 400 W led to reduced yield. The authors concluded that higher microwave power enhances protein extraction by disrupting cellular structures and facilitating protein release and recovery. EAE showed a lower increase in protein yield compared with MAE and UAE, which could be due to partial protein degradation by papain enzyme during treatment [12]. However, when compared to CE, a significant increase in yield (*p* < 0.05) was noted (14.60–21.03%). This could be due to the hemicellulolytic and pectolytic activities of viscozyme (a multi-enzyme complex), which hydrolyzes the glycolytic bonds in the hemicellulose and pectin component of the plant cell wall. This enzymatic breakdown disrupts the complex polysaccharide matrix, loosening the cell wall structure and thereby facilitating protein extraction from moringa seed.

Regardless of the extraction method used, protein constituted the predominant component of each isolate (80.47–86.61 g/100 g, d.w), with minor amounts of fat residue (0.66–1.57 g/100 g), ash (2.44–3.73 g/100 g), and moisture (2.18–4.18 g/100 g), which were comparable to those of C-SPI used as reference. The CE method recorded the lowest protein content (80.47 g/100 g), which aligns with the 80.98% reported by Illingworth et al. [13] for moringa seed protein extracted using the traditional alkaline method.

**Figure 1:**
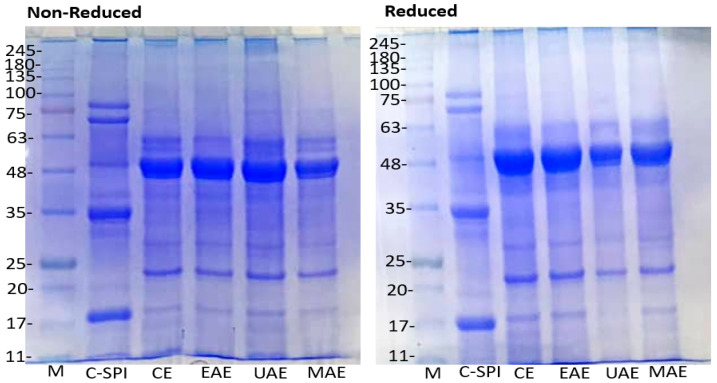
Influence of extraction method on electrophoretic profile of MSPI. M: standard protein maker, CE: alkaline extraction, EAE: enzyme-assisted extraction, UAE: ultrasonic-assisted extraction, MAE: microwave-assisted extraction, C-SPI: commercial soy protein isolate. Gel used: 12% separating gel and 4% stacking gel. A total of 15 μg protein was loaded in each lane.

All novel-assisted extraction methods produced protein isolates with purity above 80% (80.57–86.61 g/100 g), meeting the criteria for classification as protein isolates. MAE yielded the highest protein content (86.61 g/100 g), followed by UAE (83.56 g/100 g), closely comparable to C-SPI (94.01 g/100 g). The superior performance of MAE is likely due to microwave-induced rapid heating, which disrupts cell structures and hydrophobic interactions, enhancing more protein recovery. These findings are consistent with earlier studies reporting higher protein purity with MAE than UAE and EAE [14,23]. Authors attributed this enhancement to the local heating effect of microwaves, which disrupts hydrogen bonds and hydrophobic interactions, thus, facilitating the release of tightly bound proteins and improving purity [23]. Comparable levels of protein purities have also been reported in some traditional protein sources, such as pea, soy, and faba bean isolates [24,25]. On the contrary, Tang et al. [26] reported a high protein content of 93.84% for moringa seed protein extracted using the traditional alkaline method. The variation may be attributed to differences in extraction conditions and seed variety. The lack of significant improvement in protein content seen in EAE (80.57 g/100 g) compared to CE (80.47 g/100 g) could be due to partial protein hydrolysis during enzyme treatment, as indicated by the SDS-PAGE (Figure 1), which could have lowered the measurable protein content [27].

According to Codex Alimentarius [28], plant protein isolates should have a moisture content below 7% to ensure storage stability and shelf life. In this study, all MSPI samples, including C-SPI, met this standard (2.18–4.18%), indicating good storage quality and stability. Similar values were reported for pea and amaranth protein isolates extracted via UAE [19,29]. The lower moisture content in MAE samples (2.29%), compared to other novel methods, could be due to microwave-induced rapid heating and localized thermal effects which promote surface water evaporation and partial dehydration during processing [30]. Ash content was significantly lower (*p* < 0.05) in all novel-assisted extraction methods (2.03–3.34%) compared to CE. Similar reductions in ash were reported for microwave-extracted cottonseed and cowpea proteins [30,31]. EAE showed the lowest ash content among the extraction methods, possibly due to enzymatic disruption of protein–mineral complexes, which breaks the binding interactions (such as ionic, covalent, or chelation) between proteins and minerals [12]. Once these complexes are hydrolyzed, minerals that were tightly bound to the protein matrix are released and can be lost during protein precipitation and centrifugation. This results in a measurable reduction in ash content. This is consistent with the findings in whey protein treated with different enzymes [32]. Residual fat content in UAE and EAE samples were generally lower than CE (*p* > 0.05), except in MAE (1.57 g/100 g, *p* > 0.05). This is possibly due to microwave-induced disruption of fat–protein interactions and partial lipid emulsification, which may hinder complete fat separation [30].

### 3.2. Anti-Nutritional Factors (ANFs)

The primary objective of any extraction process is to maximize target yield while minimizing co-extraction of undesired components and preserving functionality. In raw moringa seed flour, trypsin inhibitor activity (TIA) ranges from 11.60 to 30.20 U/mg protein [33], while phytate content was reported as 19.29 mg/g [4]. All protein extraction methods evaluated in this study significantly (*p* < 0.05) reduced phytate (0.83–0.49 g/100 g) and TIA (4.48–1.92 U/mg), with MAE showing the greatest reduction, followed by EAE, comparable to commercial soy protein. Trypsin inhibitors consist of two main fractions which are eluted at pH 3.5 and 4.0 [34]. The reduction in TIA in our findings may result from the isoelectric precipitation of proteins at pH 4.5, which partially excluded these inhibitors. The enhanced reductions in TIA in MAE and UAE could possibly be due to the energy-induced disruption of phytate–protein and inhibitor complexes [35]. Enzymatic hydrolysis reduced TIA by possibly modifying its active sites, thereby preventing interaction with trypsin. Consistent with our findings, Sa et al. [36] showed that MAE (850 W) and UAE (40 kHz) significantly reduced phytate and TIA in pumpkin seed protein by 71.26 and 54.68%, respectively. Authors attributed these effects to acoustic cavitation, which disrupts TIA–trypsin interactions. The reported phytic acid in MSPI is lower than the levels reported in pea (1.2 g/100g) and chickpea proteins (1.5 g/100 g) [37]. Emerging evidence suggests that residual phytic acid may offer antioxidant, anti-inflammatory, and antidiabetic benefits when consumed within the safe intake range of 250–500 mg/100 g [34,38]. Phytic acid levels in the extracted moringa seed protein fall within this tolerable intake range. On the other hand, while the novel-assisted extraction methods significantly reduced TIA, the safety of direct consumption cannot be confirmed due to the absence of a universal threshold. However, for food applications, especially infant foods, Duque-Estrada et al. [39] suggested that TIA should be reduced to below ~4 TIU/mg protein to improve protein digestibility. The reduced ANFs, together with the enhanced IVPD and functional properties of MSPI, highlight its potential as a high-quality protein source. However, despite inactivation at the end of extraction process, residual enzymes can still compromise storage stability. Therefore, suitable storage conditions, such as refrigeration, vacuum sealing, or inert-atmosphere packaging, are recommended.

### 3.3. Molecular Weight Distribution by SDS-PAGE

The electrophoretic profiles of extracted proteins using novel techniques are shown in Figure 1. Under non-reducing conditions, all MSPI samples exhibited prominent bands at ~25 and 48–63 kDa, indicating a common major storage protein subunit across methods. Faint bands observed at ~75–135 kDa suggest protein aggregates or multimeric complexes stabilized by disulfide bonds. Similar patterns were reported by Tang et al. [26] and Anyiam et al. [4] who observed major bands between 17 and 63 kDa in moringa seed protein. These correspond to globulins (~50 kDa) and low-molecular-weight albumins (10–20 kDa), which are reported as the major storage proteins in *Moringa* seeds [40], supporting our findings. Upon reduction with β-mercaptoethanol (ME), the major bands at 48–63 kDa showed no alterations, indicating that these bands lack disulfide bridges, supporting previous reports [4,26]. However, the light intensity higher-molecular-weight bands (>75 kDa) disappeared under reducing conditions, while a band at ~17–20 kDa emerged, indicating the presence of disulfide-linked subunits which were broken down after the addition of ME. C-SPI shows a similar molecular weight banding pattern with distinct bands at 17, 35, 48, and 75 kDa, reflecting a comparable protein composition or structure.

The SDS-PAGE profiles of extracted proteins using the novel-assisted methods were similar to CE, except for EAE, which showed slightly reduced band intensity around ~63 kDa, indicating partial hydrolysis due to the proteolytic activities of papain enzyme. No major structural changes were observed across methods, suggesting that the extraction treatments did not alter the protein’s primary structure. This is consistent with previous findings in amaranth [19], pea [29], and jack bean proteins [14], showing no alterations in the molecular structures of proteins following the application of novel extraction methods. This may be due to the extraction methods not causing protein fragmentation, and the absence of significant proteolysis or degradation of proteins. On the contrary, Mathews et al. [22] reported protein degradation in sesame isolate under UAE and MAE, and attributed this to the removal of allergenic proteins found in sesame.

### 3.4. Effect of Extraction Methods on the Color Characteristics

Color, a key factor in consumer acceptance, was significantly influenced by the extraction methods (Table 2). Lightness (L*) slightly decreased (82.61–79.14), while yellowness (b*) increased across all novel methods compared to CE, except for EAE, which had similar b* values to C-SPI (*p* > 0.05). The whiteness index (WI) also decreased significantly (*p* < 0.05), except in EAE proteins which showed higher WI and lower b*, making it visually closest to C-SPI. The color differences between MSPI and C-SPI likely stem from variations in pigment composition, storage compounds, and phytochemicals such as phenolics [4]. Additionally, the increased yellowness observed in the MSPI can be attributed to its higher flavonoid and iron content, which are known contributors to yellowness [4,5]. Higher b* values in MAE and UAE may result from enhanced pigment release due to microwave- and ultrasound-induced thermal and cavitation effects. Similar trends were observed by Naik et al. [41] in ultrasound-extracted bitter melon seed protein, which showed reduced lightness and increased yellowness compared to CE.

The retained whiteness and lower yellowness in EAE may result from mild enzymatic conditions that limit pigment degradation and browning. This aligns with the report of Akyuz and Ersus [42] showing high brightness (79.6) and low yellowness (13.27) in sugar-beet protein isolates using EAE. The total color difference (ΔE) was used to quantify the changes in color shifts between all extraction methods compared to C-SPI. The ΔE followed the decreasing order: MAE > CE > UAE > EAE > C-SPI. Protein extracted using MAE exhibited the highest ΔE value (12.60), reflecting a major perceptible color shift. This is likely due to the formation of Maillard reaction products (e.g., melanoidins) via the reaction between reducing sugars and amino acids, driven by the dielectric heating effect of MAE. Similar results were also noted in microwave-treated sesame seeds [22] and fortail millet protein isolates [43], where the authors linked color changes to microwave-induced heating that promotes Maillard reactions and pigment degradation. EAE showed moderate color changes, suggesting that this method induced less drastic alterations in surface pigmentation.

### 3.5. Effect of Extraction Methods on Protein Solubility

**Figure 2:**
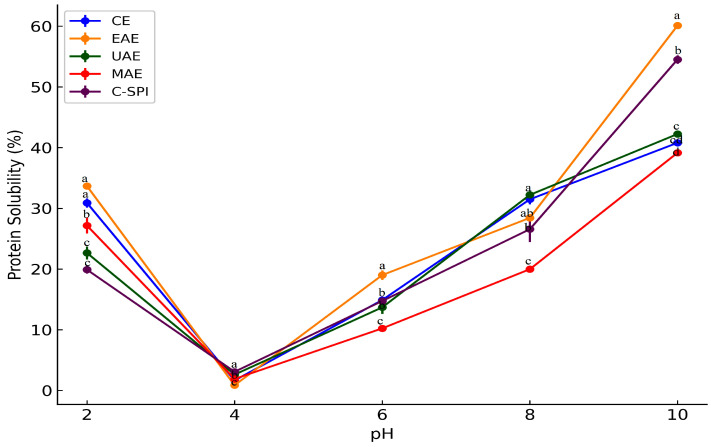
Impact of extraction method on protein solubility pattern of MSPI. Different letters indicate statistically significant differences among treatments (*p* < 0.05). CE: Alkaline extraction, EAE: enzyme-assisted extraction, UAE: ultrasonic-assisted extraction, MAE: microwave-assisted extraction.

Protein solubility is crucial for functional properties and depends strongly on pH [44]. As shown in Figure 2, MSPI solubility displayed a U-shaped curve across pH 2–10, with minimum solubility at pH 4 (near the isoelectric point) and maximum at pH 10. This pattern aligns with findings in pea, quinoa, and soy proteins [27,45] and is driven by pH-induced changes in protein charge and structure [46]. Minimal solubility near the isoelectric point results from reduced net charge and increased hydrophobic interactions, while the increase at alkaline pH (8–10) is likely due to protein unfolding and greater electrostatic repulsion, which enhance protein–water interactions [44,46]. Novel-assisted methods significantly improved protein solubility at pH 10 (40.78–60.09%), with EAE showing the highest value. This is likely due to enzymatic hydrolysis exposing hydrophilic residues, thereby promoting protein–water interactions. Similar results were reported for enzyme-treated pea, quinoa, and soy proteins [27,46,47].

UAE proteins also showed increased solubility, compared to CE, likely due to cavitation-induced surface charge exposure, partial unfolding, and reduced particle size, which enhance protein–water interactions [45]. A similar result was observed in ultrasonication-treated moringa seed protein [6], where protein solubility increased from 5.56 to 29.82% at neutral pH. The authors attributed this to the reduction in particle size and alterations in the molecular structure of proteins which expose more hydrophilic groups. However, Sharma et al. [48] reported that excessive ultrasound can reduce protein solubility through aggregation or oxidative damage, highlighting the need to optimize sonication conditions. The slight decrease in solubility observed in MAE protein, despite its higher protein purity, could be attributed to rapid localized heating that exposes more hydrophobic regions and promotes partial aggregation [7,8]. Similar observation was reported in microwave-treated proteins from pea [49], cottonseed [30], and jack bean [14]. Authors linked this effect to structural damage, sulfhydryl loss, and oxidative modifications induced by microwave heating. However, Zheng et al. [50] reported improved solubility in lotus seed protein under milder microwave conditions, highlighting the influence of microwave power and duration on protein solubility.

### 3.6. Effect of Extraction Methods on Protein Techno-Functional Properties

#### 3.6.1. Water/Oil Holding Capacities

Protein functional properties influence processing and storage performance, making it essential to evaluate extraction effects. Water and oil holding capacities (WHCs and OHCs) are critical for enhancing texture, flavor, and moisture retention in food products [14]. As shown in Table 3, all novel-assisted extraction methods significantly improved WHC and OHC compared to CE. WHC increased from 2.01 to 2.78 g/g, with EAE showing the highest value, though still below the WHC of C-SPI (6.46 g/g). This improvement likely results from enzymatic hydrolysis, which exposes hydrophilic residues and increases porosity [51]. This is consistent with the findings in coconut protein treated with alcalase enzyme [52]. Ultrasonic cavitation improved the WHC of MSPI (2.01–2.73 g/g), likely by disrupting aggregates and exposing hydrophilic and thiol groups [20]. This aligns with a previous report on quinoa proteins [45], which was attributed to reduced particle size and increased surface area following ultrasound treatment. MAE showed the lowest increase in WHC (2.01–2.68 g/g) among the novel extraction methods, which could be attributed to rapid and uneven heating, limiting structural changes and hydrophilic site exposure. Microwave treatment has been shown to enhance the WHC of plant proteins in previous studies. For example, Mathews et al. [22] reported an increase in the WHC of sesame protein from 1.33 to 2.16 g/g, while Coutinho et al. [11] observed a similar improvement (1.65–1.88 g/g) in pea protein following MAE at 900 W for 120 s. These enhancements were attributed to structural modifications that increased the ability of the protein to retain water.

On the contrary, Ajayi et al. [14] reported higher improvement in WHC, from 1.4 to 2.7 g/g, in microwave-extracted jack bean protein than in ultrasound-treated samples (1.4–2.0 g/g). The variation is likely due to the differences in protein source and extraction conditions. Proteins with WHC values between 1.49 and 4.72 g/g are suitable for moisture-sensitive products such as baked goods and meat substitutes [29], supporting the suitability of MSPI in such applications. OHC reflects the ability of protein to bind with oil, and it is essential for flavor retention. The OHC values of proteins extracted using novel methods (2.52–2.67 g/g) were similar to that of C-SPI and significantly exceeded the control (CE) (2.11 g/g). MAE showed the highest OHC (2.67 g/g), likely due to microwave-induced unfolding that exposed more hydrophobic residues, which enhances oil binding [11]. This aligns with previous studies reporting improved OHC in plant proteins after microwave treatment due to structural changes [22,43]. However, Kadam et al. [30] observed a decline in the OHC of microwave-treated cottonseed protein, which was attributed to steric hindrance from excessive hydrophobic exposure. EAE and UAE showed improved OHCs which were comparable to C-SPI. Similar improvements were observed in pecan [27] and quinoa proteins [45] after enzymatic and ultrasonic treatments. This could be attributed to enzymatic hydrolysis which breaks down protein chains exposing some initially buried hydrophobic groups and also ultrasound which disrupts protein aggregates and alters structure, both increasing surface activity and oil-binding capacity. Enhanced OHCs indicate that the novel-extracted MSPIs (particularly, MAE) are suitable for fat-retentive foods like baked goods, meat analogs, and spreads.

#### 3.6.2. Foaming Properties

Foaming capacity (FC) is the ability of proteins to quickly unfold and form a cohesive layer around gas bubbles, while foaming stability (FS) indicates the ability of a substance to sustain foam over time by forming a cohesive intermolecular network that traps and supports air bubbles [20]. All novel extraction methods significantly improved FC and FS values of MSPIs (*p* < 0.05) (Table 3), with enzyme-assisted extraction showing the highest values. This is likely due to improved protein solubility and exposure of hydrophilic residues, which enhance interfacial adsorption and air incorporation. This result is consistent with previous reports in the literature. For instance, Yang et al. [45] reported 1.43- and 1.44-fold increases in FC and FS, respectively, in quinoa protein extracted using UAE and EAE methods. Similarly, Wang et al. [29] observed improvements in FC (69–82%) and FS (77–95%) in pea proteins extracted using ultrasonic techniques. The improved FC and FS in protein treated with microwaves may result from partial protein unfolding and increased surface hydrophobicity, which enhances air–water interface adsorption [11]. Similar improvements in foaming capacity were observed in sesame and jack bean proteins treated with MAE [14,22].

On the contrary, Rao et al. [53] reported reduced FC in millet protein treated at a microwave power of 784 W for 4 min. This was attributed to rapid local overheating of the microwave treatment, which causes protein denaturation and the formation of insoluble aggregates that hinder protein adsorption at the air–water interface. The variations could be due to differences in protein type, microwave power, and treatment duration. The improved FC in ultrasound-treated MSPI (27.11–36.77%) aligns with previous findings for pea and quinoa proteins [29,45], which was attributed to reduced particle size and partial unfolding from ultrasonic treatment. The lack of a significant result in FS of MSPI after ultrasonication (*p* > 0.05) may be attributed to reduced surface activity caused by the loss of intermolecular cohesiveness and elasticity. This reduction in surface activity weakens the ability of the protein to adsorb and properly orient at the air–water interface, limiting the formation of a stable viscoelastic protein film around air bubbles, and resulting in poor stabilization of foams and ultimately a reduction in foaming capacity and stability [7]. This is consistent with previous findings showing no significant change (*p* > 0.05) in the foaming stability of moringa seed protein after ultrasonication across ultrasound powers of 20–100% [26]. The enhanced foaming properties of MSPIs suggest their suitability for potential applications in food products such as ice cream, baked goods, and desserts.

#### 3.6.3. Emulsifying Properties

Proteins are amphiphilic (containing both hydrophilic and hydrophobic regions), which enable them to adsorb at the oil–water interface and function as effective food emulsifiers [10]. The emulsifying activity index (EAI) of MSPI significantly increased (50.46–65.90 m^2^/g; *p* < 0.05), while the emulsion stability index (ESI) decreased (37.87–22.98%; *p* < 0.05) across all novel extraction methods (Table 3). MAE showed the highest EAI (65.90 m^2^/g) followed by UAE (56.80 m^2^/g) but both recorded a lower ESI than CE. The enhanced EAI is possibly due to protein unfolding, increased surface hydrophobicity, and reduced interfacial tension [22]. Similar improvement was reported in pigeon pea, soy, and quinoa proteins following microwave and ultrasound treatments [11,45,54]. EAE recorded the lowest improvement in the EAI (50.46–53.70 m^2^/g) among the novel methods, possibly due to the formation of smaller and more soluble peptides with greater flexibility and faster diffusion to the oil–water interface [8,9]. Although emulsifying properties were thought to be associated with increased surface hydrophobicity, recent studies suggest that protein structural flexibility may play a more decisive role [10,54]. Consistent with our results, previous reports found that enzyme-extracted pea, soy, and pecan protein isolates exhibited higher EAIs [27,45,54], which were attributed to the observed enhanced solubility and interfacial diffusion even though they also showed lower surface hydrophobicity, similar to our findings.

The slight reduction in the ESI observed across all novel-assisted extraction methods compared to the CE may be attributed to structural disruptions caused by these extraction methods. According to Ajayi et al. [14], ultrasonication can lead to protein unfolding and partial aggregation, thus, reducing interfacial film strength. On the other hand, uneven heating during MAE could disrupt protein conformation, causing denaturation or aggregation [8]. This weakens interfacial film formation, leading to reduced ESI. A similar reduction in the ESI was observed by Constantino and Garcia-Rojas [19] in microwave-treated amaranth protein isolate, where reduced emulsion stability was linked to excessive protein unfolding followed by re-aggregation, which compromised the integrity of the interfacial film. Nonetheless, the enhanced emulsifying activity of MSPI indicates strong potential for use in emulsion-based food products such as sausages and mayonnaise.

#### 3.6.4. Surface Hydrophobicity

Protein surface hydrophobicity (Ho), an indicator of exposed hydrophobic groups and conformational changes, is closely linked to protein functionality [20]. As shown in Table 3, UAE and MAE significantly increased Ho (69.91–87.12 μg; *p* < 0.05), with MAE showing the highest value. In contrast, EAE significantly reduced Ho (69.91–38.26 μg) compared to CE. The increase in Ho of the MSPI after microwaving could be attributed to microwave-induced spatial polarization, which disrupts non-covalent interactions within the protein structure, leading to the exposure of buried hydrophobic residues [7,45]. A similar improvement in Ho (45.04–104.22 μg) was reported by Ajayi et al. [55] for microwave-extracted jack bean protein at similar conditions of 800 W, compared to UAE and CE control. The enhancement in Ho after sonication was attributed to the cavitation effects of ultrasound on the protein structure. Under ultrasonic cavitation, the complex structure of the protein is disrupted as the external physical forces loosen its molecular conformation. This disruption likely causes the release of hydrophobic amino acid residues that were originally buried inside the molecules [27]. The exposure of these residues may explain the increased surface hydrophobicity observed in the protein extracted using the ultrasound-assisted method compared to the control. Hegde et al. [20] reported a similar significant increase (81.34–92.40 μg) in the Ho of ultrasonicated pearl millet protein isolate, which aligns with our findings. The decrease in the Ho of MSPI following EAE may be due to the generation of smaller peptides with lower surface hydrophobicity. Another possible explanation is that viscozyme breaks down the biomass cell wall, exposing hydrophobic regions on the protein surface that are subsequently hydrolyzed by papain. This process reduces the number of intact hydrophobic domains available for surface interactions, thereby lowering the overall Ho of the protein. Similarly to our findings, Yang et al. [45] and Wang et al. [27] reported that enzyme-extracted pea and pecan protein isolates exhibited lower surface hydrophobicity than those extracted via ultrasound. In contrast, Jiang et al. [21] reported a 39.95% increase in the Ho of *Akebia trifoliata* protein isolate following EAE using cellulase, while UAE led to a 43.12% reduction in the Ho. The authors attributed the increase in Ho to the degradation of the cellulose-rich cell wall by cellulase, which exposed more hydrophobic groups on the protein surface. In contrast, the reduction in Ho after UAE was linked to the excessive cavitation effects, leading to protein unfolding and subsequent refolding, causing aggregation that likely concealed these hydrophobic residues and reduced Ho [21]. The differences may be due to the specific enzyme used, as well as variations in ultrasound conditions and duration of treatment. The increased Ho observed in MAE in our study may account for its enhanced OHC and EAI compared to other extraction methods.

### 3.7. Influence of Extraction Methods on Thermal Stability

**Figure 3:**
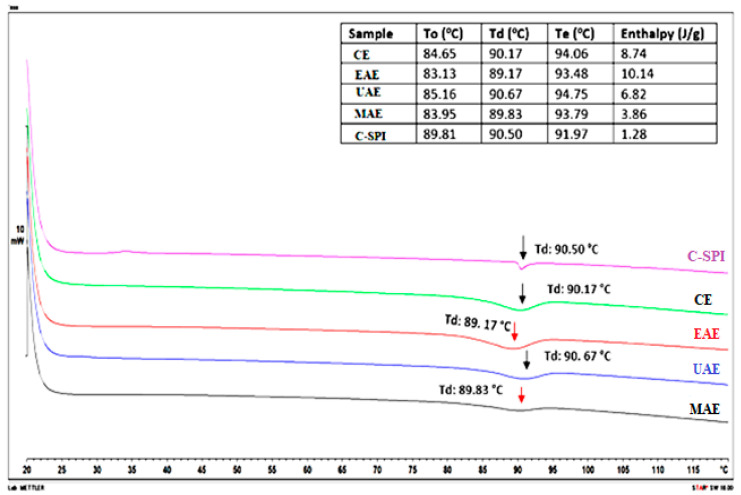
Effect of different novel-assisted extraction methods on thermal properties of MSPI. C-SPI: commercial soybean protein isolate, CE: alkaline extraction, EAE: enzyme-assisted extraction, UAE: ultrasonic-assisted extraction, MAE: microwave-assisted extraction. To: Onset temperature, Td: denaturation temperature, Te: end temperature.

The thermal stability of proteins represents its resistance against aggregation during heating [48]. It is measured with the help of differential scanning calorimetry (DSC). In the DSC thermogram, the temperature corresponding to the maximum peak represents the denaturation temperature (Td) at which protein denaturation occurs, while the transition enthalpy (ΔH) indicates the total heat energy required for denaturation and reflects changes in the ordered structure of proteins [20,27]. As shown in Figure 3, MSPI samples exhibited slight variations in Td and ΔH across extraction methods. The UAE-treated MSPI showed a slight increase in Td (albeit, not significant). This is attributed to ultrasound-induced structural rearrangement enhancing thermal stability. Ultrasonic cavitation produces intense shear forces, causing partial unfolding and reorganization of protein molecules [7]. This could promote stronger intramolecular interactions, such as hydrogen bonding and hydrophobic interactions, which stabilize the protein’s tertiary structure and which may explain the slight increase in Td. Our finding is consistent with results reported for sesame protein extracted using UAE [22], where Td slightly increased by 8.8% compared to conventional extraction methods. In contrast, MAE and EAE slightly reduced Td to 89.83 °C and 89.17 °C, respectively, likely due to thermal disruption and partial enzymatic hydrolysis. A similar, non-significant reduction (92.2–91.7 °C) in Td was reported in pigeon pea protein treated with microwaves at 700 W for 120 s [11]. This indicates that the core structural stability of the proteins remains unaffected by the mechanical treatments. The lower Td observed in enzyme-treated MSPI also aligns with its partial hydrolysis, suggesting reduced structural integrity and thermal stability.

Transition enthalpy reflects the energy required to disrupt interactions stabilizing protein tertiary structures, making it a key indicator of protein denaturation. A progressive decline in ΔH was observed for UAE (8.74–6.82 J/g) and MAE (8.74–3.86 J/g), indicating partial protein unfolding and reduced structural integrity, requiring less energy for denaturation. Notably, MAE yielded the lowest ΔH (3.86 J/g), approaching that of commercial soy protein (ΔH: 1.28 J/g; Td: 90.50 °C). In contrast, the higher transition enthalpy seen in EAE (8.74–10.14 J/g) suggests a more preserved native state, requiring more heat in the unfolding of the protein structures. This implies that MSPI may exhibit similar heat stability with C-SPI during food processing, making it suitable alternatives to soy protein in high-temperature applications, such as baked goods, meat analogs, and plant-based dairy products, which involves high thermal processing.

### 3.8. In Vitro Protein Digestibility (IVPD)

**Figure 4:**
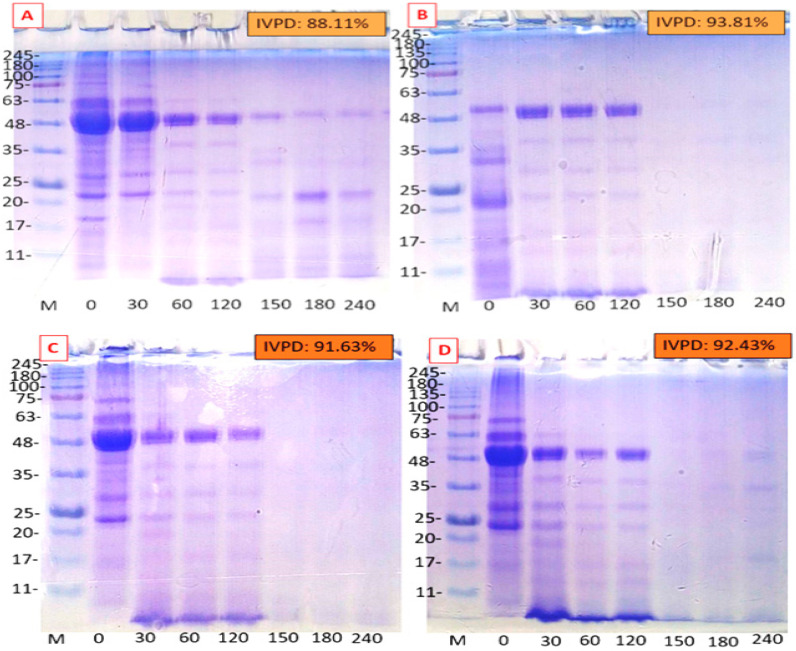
Influence of extraction technique on in vitro protein digestibility pattern of MSPI. (**A**): Alkaline extraction, (**B**): enzyme-assisted extraction, (**C**): ultrasonic-assisted extraction, (**D**): microwave-assisted extraction. IVPD: in vitro protein digestibility. Gel used: 12% separating gel and 4% stacking gel. 15 μg protein was loaded in each lane.

Protein digestibility of plant sources is crucial for food applications as it reflects how efficiently ingested protein is utilized. The IVPD of MSPIs ranged from 88.11 to 93.81% across the novel extraction methods (Figure 4). EAE had the highest IVPD, closely matching that of C-SPI (94.01%) and significantly higher than the conventional method (*p* < 0.05). The digestibility order was C-SPI > EAE > MAE > UAE > CE. In our previous study, moringa seed whole flour showed an IVPD of 83.19% in raw form [4]. The higher digestibility observed in this study using novel-assisted extraction methods is likely due to the enhancement in protein purity, which concentrates the protein and reduces ANFs, thereby improving enzyme accessibility during digestion. Similarly, Mathews et al. [22] found that MAE improved sesame protein digestibility (89.57–94.44%) more than UAE and the alkaline method. The high IVPD of enzyme-treated MSPI may result from papain-induced hydrolysis, enhanced solubility, and better enzyme access. This is consistent with Kim et al. [56], who showed similar effects in enzyme-treated pea protein. MAE enhanced the IVPD (92.45%) of MSPI more effectively than UAE (91.63%), despite a notable drop in protein solubility, compared to CE. Although protein solubility may correlate positively with digestibility [57], present findings align with studies showing that digestibility can improve due to structural changes in protein and reduced ANFs. For example, Sun et al. [49] and Karki et al. [43] reported increased IVPDs in pea and foxtail millet proteins after microwave-assisted extraction, despite the reduced solubility observed. This was attributed to enhanced protein purity and structural denaturation. These results suggest that conformational changes, reductions in ANFs, and increased enzyme accessibility likely contributed to the improved IVPD observed.

The lowest increase in IVPD recorded in UAE compared to CE (88.11–91.63%) may be due to limited cleavage site exposure from mild acoustic cavitation. This aligns with Sun et al. [49] and Loushigam & Shanmugam [31] who reported a minimal IVPD increase in sonicated pea protein, which was attributed to the short sonication time applied. Jin et al. [58] further noted that while moderate ultrasound improved buckwheat protein digestibility, excessive intensity reduced it due to aggregation, highlighting the need to optimize sonication parameters. The lower IVPD observed in traditionally extracted MSPI (88.11%) may be linked to residual impurities and anti-nutritional factors. SDS-PAGE analysis corroborates these findings, showing a progressive breakdown of major protein bands (48–63 kDa) during digestion, with near-complete degradation after 240 min across the novel-assisted extraction methods indicating enhanced protein digestibility.

### 3.9. Changes in Structural Morphology

**Figure 5:**
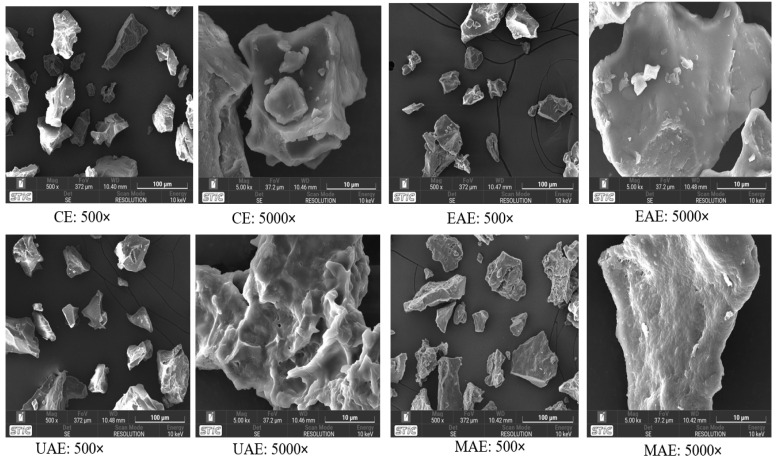
Scanning electron microscopy characteristics of moringa seed protein isolate. CE: Alkaline extraction, EAE: enzyme-assisted, UAE: ultrasonic-assisted, MAE: microwave-assisted. 500× and 5000×: Magnifications.

SEM analysis showed distinct surface morphologies of MSPI across extraction methods at 500× and 5000× magnifications (Figure 5). CE protein appeared compact and dense with minimal porosity, reflecting limited matrix disruption and strong protein–protein interactions similar to the high-pH extraction previously reported in rice bran protein [48]. EAE displayed multiple irregularly shaped particles with angular appearances and relatively smooth surfaces, along with some visible micro-cracks at higher magnification, indicating partial hydrolysis and loosening of the matrix by enzymatic action. This morphological change exposes more surface area that can enhance the accessibility of digestive enzymes, which in turn could further contribute to the improved digestibility observed in this study. A similar morphological pattern was observed by Yang et al. [45] and Yeasmin et al. [12] in quinoa and *Phaseolus vulgaris* proteins, respectively, after enzymatic treatment. The authors attributed this to greater structural degradation and the proteolytic action of the enzymes, which was linked to the higher emulsification activity and digestibility [12]. UAE caused the most extensive structural breakdown, producing porous, fragmented, and sponge-like particles due to acoustic cavitation-induced cell wall rupture. As a result, the accelerated protein extraction yield recorded for UAE in this study could be attributed to the alterations in the microstructural arrangement of proteins induced by ultrasonication. Similar SEM images were reported in rice bran and cowpea proteins after ultrasound treatment [31,48]. MAE showed broad and cracked structures possibly due to rapid heating and moisture expansion, which likely promotes disulfide bonding and β-sheet formation, which resulted in observed slight reduction in solubility. This is consistent with findings in pea and jack bean proteins [49,55]. However, such structural changes seen in MAE may also expose enzymatic cleavage sites and enhance protein digestibility [14]. Although this study did not examine how extraction methods affect gelation properties of MSPI, Ajayi et al. [14] suggested that such microwave-induced aggregates as observed in SEM images can also improve gelation by strengthening the protein network. According to Yang et al. [45], the microstructure of a protein can influence its physico-chemical properties, including functionality. Therefore, it is reasonable to suggest that the morphological characteristics of MSPI extracted using different novel technologies contributed to the enhancement of its techno-functional properties and digestibility.

### 3.10. Comparative Radar Plot of MSPIs

**Figure 6:**
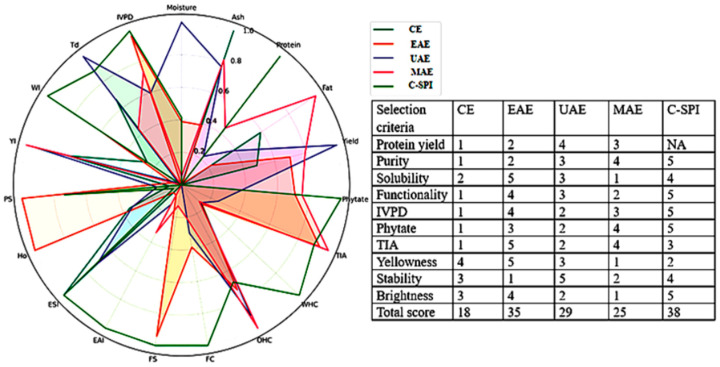
Comparative radar analysis of MSPIs extracted using different methods. CE: alkaline extraction, EAE: enzyme-extraction, UAE: ultrasonic extraction, MAE: microwave extraction.

The radar plot and scoring table which compare the performance of MSPIs extracted via different methods across multiple criteria are shown in Figure 6. The C-SPI used as benchmark achieved the highest total score of 38, followed closely by EAE with a total of 35. The overall ranking based on total scores was C-SPI > EAE > UAE > MAE > CE. EAE demonstrated superior performance in key properties, such as protein solubility, IVPD, functional properties, and reduction in ANFs (i.e., phytate and TIA). It also scored highest in color quality, reflecting better visual appeal. UAE achieved the highest score in protein yield and thermal stability, while MAE had a high protein purity score but scored lowest on solubility and color attributes. The control sample (CE) consistently showed the lowest scores across most parameters.

## 4. Conclusions

This study provides valuable comparative insights into the effects of different novel-assisted extraction techniques on the techno-functional and nutritional properties of moringa seed protein isolates. Among the methods, EAE produced protein isolates with superior solubility, digestibility, and the highest reduction in anti-nutritional factors. UAE achieved the highest extraction yield and thermal stability, while MAE recorded the highest protein content, surface hydrophobicity, and emulsifying capacity, though with a slight reduction in solubility compared to CE. Overall, UAE and MAE are most effective when protein quantity is prioritized, offering higher yield and purity, respectively. EAE is best suited for applications requiring improved functional and nutritional qualities of moringa seed protein, such as in protein-enriched beverages and food formulations. Therefore, the choice of extraction method should be guided by the intended application of the protein in food systems, while also taking the economic feasibility into account for commercial production. Future studies should focus on optimizing these extraction conditions, characterizing effects on amino acid profiles and bioactive properties, and assessing the performance of MSPIs in various food applications to support their potential for industrial use.

## Figures and Tables

**Table 1:** Composition, yield, and anti-nutrients of MSPIs obtained by different extraction methods.

Sample	Moisture	Ash	Protein	Fat	Yield (%)	Recovery Rate (%)	Phytate (g/100 g)	TIA (TIU/mg Protein)
CE	2.18 ± 0.10 ^c^	3.73 ± 0.72 ^a^	80.47 ± 0.42 ^d^	1.09 ± 0.24 ^b^	14.60 ± 1.13 ^d^	37.47 ± 4.25 ^d^	0.83 ± 0.04 ^a^	4.48 ± 0.69 ^a^
EAE	2.96 ± 0.32 ^b^	2.44 ± 0.45 ^b^	80.57 ± 0.38 ^d^	0.66 ± 0.21 ^bc^	21.03 ± 0.75 ^c^	55.85 ± 1.74 ^c^	0.51 ± 0.02 ^c^	2.10 ± 0.28 ^c^
UAE	4.18 ± 0.56 ^a^	3.24 ± 0.11 ^a^	83.56 ± 0.50 ^b^	0.85 ± 0.06 ^bc^	30.08 ± 2.60 ^a^	78.43 ± 3.93 ^a^	0.66 ± 0.14 ^b^	3.85 ± 0.29 ^a^
MAE	2.29 ± 0.16 ^bc^	3.34 ± 0.12 ^a^	86.61 ± 0.80 ^c^	1.57 ± 0.38 ^a^	23.94 ± 8.80 ^b^	66.06 ± 2.31 ^b^	0.49 ± 0.13 ^c^	1.92 ± 0.06 ^c^
C-SPI	3.01 ± 0.78 ^b^	1.62 ± 0.05 ^c^	94.30 ± 2.72 ^a^	0.40 ± 0.04 ^d^	NA	NA	0.38 ± 0.03 ^d^	2.19 ± 0.33 ^b^

Data is expressed as mean ± standard deviation of triplicate determinations. Different letters in the same column indicate statistically significant differences (*p* < 0.05). CE: Alkaline extraction, EAE: enzyme-assisted extraction, UAE: ultrasonic-assisted extraction, MAE: microwave-assisted extraction. C-SPI: Commercial soy protein isolate. TIA: Trypsin inhibitor activity. NA: Not applicable.

**Table 2:** Impact of different novel-assisted extraction techniques on color characteristics of MSPIs.

Sample	Appearance	L*	a*	b*	YI	WI	ΔE
CE	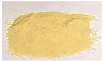	82.61 ± 0.14 ^b^	2.63 ± 0.25 ^a^	22.68 ± 0.22 ^b^	39.22 ± 0.32 ^b^	71.29 ± 0.07 ^c^	11.14 ± 3.52 ^a^
EAE	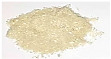	80.32 ± 0.19 ^c^	0.38 ± 0.15 ^b^	12.01 ± 0.18 ^c^	21.37 ± 0.30 ^c^	76.94 ± 0.14 ^b^	4.18 ± 0.41 ^b^
UAE	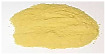	79.14 ± 0.27 ^e^	2.83 ± 0.35 ^a^	25.00 ± 0.42 ^a^	45.13 ± 0.88 ^a^	67.31 ± 0.46 ^d^	4.33 ± 5.91 ^b^
MAE	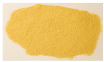	79.83 ± 0.10 ^d^	3.04 ± 0.47 ^a^	25.97 ± 2.47 ^a^	46.48 ± 4.48 ^a^	66.95 ± 2.05 ^d^	12.60 ± 7.58 ^a^
C-SPI	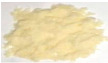	89.69 ± 0.20 ^a^	0.64 ± 0.09 ^c^	12.44 ± 0.09 ^e^	19.81 ± 0.19 ^d^	83.83 ± 0.20 ^a^	NA

Data is expressed as mean ± standard deviation of triplicate determinations. Different letters in the same column indicate statistically significant differences among different treatments (*p* < 0.05). YI: yellowness index, WI: whiteness index, ΔE: change in color, C-SPI: commercial soy protein isolate, CE: alkaline extraction, EAE: enzyme-assisted extraction, UAE: ultrasonic-assisted extraction, MAE: microwave-assisted extraction. NA: Not applicable.

**Table 3:** Effect of extraction methods on functional properties and hydrophobicity (Ho) of MSPIs.

Sample	WHC (g/g)	OHC (g/g)	FC (%)	FS (%)	EAI (m^2^/g)	ESI (%)	Ho (BPB Bound, μg)
CE	2.01 ± 0.05 ^c^	2.11 ± 0.02 ^c^	27.11 ± 0.77 ^e^	37.01 ± 0.86b ^c^	50.46 ± 1.26 ^e^	37.87 ± 1.04 ^a^	69.91 ± 2.20 ^c^
EAE	2.78 ± 0.17 ^b^	2.52 ± 0.11 ^b^	39.60 ± 1.57 ^b^	64.10 ± 4.42 ^a^	53.70 ± 0.25 ^d^	24.02 ± 0.87 ^c^	38.26 ± 1.03 ^d^
UAE	2.73 ± 0.04 ^b^	2.62 ± 0.17 ^a^	36.77 ± 0.19 ^c^	36.90 ± 0.94 ^c^	56.80 ± 1.70 ^c^	33.63 ± 1.89 ^b^	70.74 ± 0.36 ^a^
MAE	2.68 ± 0.01 ^b^	2.67 ± 0.02 ^a^	33.88 ± 0.96 ^d^	40.66 ± 1.15 ^b^	65.90 ± 0.78 ^b^	22.98 ± 0.40 ^c^	87.12 ± 0.80 ^c^
C-SPI	6.46 ± 0.29 ^a^	2.49 ± 0.16 ^b^	59.33 ± 1.15 ^a^	65.77 ± 1.53 ^a^	96.91 ± 1.08 ^a^	37.67 ± 1.04 ^a^	84.42 ± 0.65 ^b^

Data is expressed as mean ± standard deviation of triplicate determination. Different letters in the same column indicate statistically significant differences among treatments (*p* < 0.05). CE: Alkaline extraction, EAE: enzyme-assisted extraction, UAE: ultrasonic-assisted extraction, MAE: microwave-assisted extraction, C-SPI: commercial soy protein isolate. Ho: surface hydrophobicity. WHC: water holding capacity, OHC: oil holding capacity, FC: foam capacity, FS: foam stability, EAI: emulsification activity index, ESI: emulsification stability index.

## Data Availability

The original contributions presented in this study are included in the article. Further inquiries can be directed to the corresponding author.
